# Development and Validation of an Interpretable Machine Learning Model to Predict Mortality in Patients With Sepsis-Induced Coagulopathy: Multicenter Cohort Study

**DOI:** 10.2196/90285

**Published:** 2026-09-21

**Authors:** Jiaxuan Sun, Jingyuan Wang, Yuxin Dong, Jieyu Liu, Yiyun Ding, Shilin Fan, Dongyue Chen, Songtao Shou

**Affiliations:** 1 Department of Emergency, Tianjin First Central Hospital, Tianjin Medical University General Hospital, Tianjin, China; 2 Department of Emergency, Tianjin Medical University General Hospital, No. 154 Anshan Street, Heping District, Tianjin, China, 86 18834895562; 3 Department of Gastroenterology, Tianjin Medical University General Hospital, Tianjin, China; 4 Department of Critical Care Medicine, Tianjin Medical University General Hospital, Tianjin, China; 5 Department of Hepatobiliary Surgery, Tianjin Medical University General Hospital, Tianjin, China

**Keywords:** sepsis-induced coagulopathy, sepsis, machine learning, prognosis, Shapley additive explanations, SHAP

## Abstract

**Background:**

Sepsis-induced coagulopathy (SIC) is a common and severe complication in patients with sepsis, characterized by microvascular thrombosis, systemic endothelial damage, and markedly increased short-term mortality. Existing traditional clinical risk scoring systems demonstrate limited accuracy and fail to capture complex, nonlinear physiological interactions, underscoring the urgent need for advanced prognostic tools.

**Objective:**

The objective of this study was to develop and validate an interpretable machine learning (ML) model using large-scale, multicenter databases to predict early mortality in intensive care unit (ICU) patients with SIC and to evaluate its predictive performance and clinical utility compared with traditional clinical risk scores.

**Methods:**

The study retrospectively analyzed clinical data of patients with SIC from the Medical Information Mart for Intensive Care IV (MIMIC-IV), the eICU Collaborative Research Database (eICU-CRD), and Tianjin Medical University General Hospital. Feature selection was performed using LASSO (least absolute shrinkage and selection operator) regression, the Boruta algorithm, and recursive feature elimination with cross-validation, combined with multivariable logistic regression. Twelve ML algorithms were trained and compared with 4 traditional clinical scoring systems (Sequential Organ Failure Assessment, Acute Physiology and Chronic Health Evaluation II, Simplified Acute Physiology Score II, and Oxford Acute Severity of Illness Score) to predict 28-day mortality after ICU admission. Model discrimination, calibration, and clinical utility were assessed using the area under the curve (AUC), calibration curves, and decision curve analysis. Furthermore, Shapley additive explanations (SHAP) values were used to ensure model interpretability and to identify individual pathophysiological drivers.

**Results:**

A total of 7980 (66.3%) patients with SIC from the MIMIC-IV database, 3815 (31.7%) from the eICU-CRD database, and 235 (2.0%) from Tianjin Medical University General Hospital were included in the study. Ten independent predictors were identified to construct the model. The XGBoost (extreme gradient boosting) model performed the best, with an AUC of 0.899 (95% CI 0.889‐0.909) in the internal validation set and 0.882 and 0.904 in the 2 external validation sets, significantly outperforming traditional scoring systems and demonstrating higher clinical net benefit. SHAP analysis identified that the top 5 critical features were anion gap, red blood cell distribution width, lactate, total bilirubin, and age. Furthermore, an easy-to-use online tool, SIC Predict Streamlit, was developed based on this model to enable clinicians to quickly assess patient risk.

**Conclusions:**

The ML model developed in this study demonstrated superior accuracy, robustness, and generalizability in predicting early mortality in patients with SIC. The model outperformed traditional clinical scoring tools, and the findings provide valuable insights for prognostic assessment and the individualized management of patients with SIC.

## Introduction

Sepsis is a clinical syndrome associated with severe infection, characterized by life-threatening organ dysfunction resulting from a dysregulated host response to infection [1]. According to the Global Burden of Disease Study, the global age-standardized incidence rate of sepsis in 2017 was 677.5 (535.7‐876.1) cases per 100,000 population (0.7%). Sepsis-related deaths account for approximately 20% of all global deaths, making it one of the leading threats to human life [2]. Sepsis-induced coagulopathy (SIC) is characterized by vascular endothelial injury and coagulation dysfunction induced by sepsis [3]. Globally, SIC occurs in 24.0% to 60.0% of patients with sepsis; if not managed properly, it may progress to disseminated intravascular coagulation, increasing the mortality rate by up to 2-fold [4,5].

In 2017, the International Society on Thrombosis and Haemostasis (ISTH) established specific criteria and a scoring system for SIC, which primarily evaluates the condition based on the degree of thrombocytopenia, the international normalized ratio (INR), and the Sequential Organ Failure Assessment (SOFA) score. SIC is defined as a total score ≥4, and patients with a score of ≥4 have a mortality rate exceeding 30% [6,7]. These studies highlight the importance of identifying high-risk populations for SIC and implementing timely interventions. Currently, developing effective tools to accurately assess the prognosis of SIC and dynamically optimize treatment strategies has become an urgent priority for clinicians.

The development of machine learning (ML) has opened up new opportunities for clinical medical research [8]. Compared to traditional statistical models, such as logistic regression or Cox proportional hazards models, ML techniques have demonstrated superior performance in disease prediction and prognosis [9,10]. However, there remains significant room for improvement in the existing studies regarding the application of ML to predict outcomes in SIC [11-13]. These studies exhibit specific methodological limitations and suboptimal predictive performance. Specifically, models developed by Lu et al [11] and Liu et al [13] achieved moderate area under the curve (AUC) values (0.81 and 0.795, respectively) but were constrained by single-center designs and a complete lack of external validation. Although Zhou et al [12] developed an externally validated extreme gradient boosting (XGBoost) model with an internal AUC of 0.828, their study lacked essential clinical utility assessments, such as decision curve analysis (DCA), to confirm actual clinical net benefit. In light of these limitations, this study aims to construct a novel prediction model to improve predictive accuracy and enhance the overall reliability and robustness of the research findings.

Therefore, the primary aim of this study is to develop and validate a high-performance, interpretable ML model for the early prediction of 28-day mortality in patients with SIC. We leveraged and compared 12 different ML algorithms using diverse, large-scale multicenter datasets, including the Medical Information Mart for Intensive Care IV (MIMIC-IV), the eICU Collaborative Research Database (eICU-CRD), and electronic medical records from Tianjin Medical University General Hospital. We hypothesize that optimized ML algorithms can effectively capture the complex, nonlinear pathophysiological trajectories of SIC, thereby significantly outperforming traditional clinical risk assessment models—such as SOFA, Acute Physiology and Chronic Health Evaluation II (APACHE II), Simplified Acute Physiology Score II (SAPS II), and Oxford Acute Severity of Illness Score (OASIS)—in prognostic accuracy. Additionally, Shapley additive explanations (SHAP) was used to visualize feature contributions, thereby enhancing the model’s transparency and providing clinicians with actionable insights to guide early, individualized interventions and optimize clinical resource allocation [14].

## Methods

### Study Design

This study was conducted and reported in strict accordance with the Transparent Reporting of a Multivariable Prediction Model for Individual Prognosis or Diagnosis for Artificial Intelligence (TRIPOD+AI) 2024 guidelines (Checklist 1).

### Data Source

A multicenter retrospective study was conducted using deidentified data from the MIMIC-IV (v3.1) database [15], the eICU-CRD (v2.0) database [16], and the emergency intensive care unit (EICU) data from Tianjin Medical University General Hospital. The MIMIC-IV database includes hospitalization records from 2008 to 2019 at the Beth Israel Deaconess Medical Center in Boston, Massachusetts. It contains data from over 65,000 intensive care unit (ICU) admissions and more than 200,000 emergency department visits. The eICU-CRD is a multicenter database containing data on over 200,000 ICU admissions across the United States between 2014 and 2015. One of the authors, JS, completed the Collaborative Institutional Training Initiative program (Certification Number: 64628900), signed the data use agreement, and obtained access to both the MIMIC-IV and eICU-CRD databases. Additionally, patients with SIC admitted to the EICU of Tianjin Medical University General Hospital between October 2021 and December 2023 with complete data were retrospectively included as an external validation cohort.

### Ethical Considerations

This study was approved by the Ethics Committee of Tianjin Medical University General Hospital (approval number: IRB2023-YX-045‐01). Written informed consent was waived due to the retrospective nature of the study.

### Participants

Patients were included if they met the following criteria: (1) first-time ICU admission (patients with previous ICU stays and potential prior SIC episodes were systematically excluded from the analysis), and (2) patients with sepsis who were diagnosed with SIC within 24 hours of ICU admission. Sepsis was defined according to the Third International Consensus Definitions for Sepsis and Septic Shock (Sepsis-3), which includes patients with confirmed or suspected infection and a SOFA total score ≥2 [1]. Suspected infection was defined as the administration of antibiotics within 3 days of culture specimen collection. SIC was defined according to the ISTH criteria [6]. The diagnosis of SIC in patients with sepsis was established based on the best clinical and laboratory values (including platelet count and INR) obtained within the first 24 hours after ICU admission, ensuring rigorous patient selection. Detailed diagnostic criteria for SIC are provided in Table S1 in Multimedia Appendix 1.

The exclusion criteria were as follows: (1) age less than 18 years; (2) ICU length of stay less than 24 hours; (3) receipt of anticoagulation therapy prior to ICU admission; (4) history of primary coagulation disorders, hemophilia, or immune thrombocytopenia; (5) history of malignancy; (6) history of cirrhosis; (7) pregnancy; and (8) missing clinical data or incomplete laboratory records.

### Variable Extraction

PostgreSQL (version 17.6; PostgreSQL Global Development Group) and R (version 4.5.1; R Foundation for Statistical Computing) were used for data extraction and processing. Data extracted from the MIMIC-IV database included the following: (1) demographic characteristics: age, gender, height, and weight; (2) vital signs: heart rate, respiratory rate, temperature, oxygen saturation (SpO_2_), noninvasive systolic and diastolic blood pressure, and mean arterial pressure; (3) clinical risk assessment models: SOFA [17], APACHE II [18], SAPS II [19], and OASIS [20]; (4) laboratory tests: lymphocytes, white blood cells, hemoglobin, platelet count, red blood cell distribution width (RDW), neutrophils, albumin, anion gap (AG), total calcium, potassium, sodium, glucose, lactate, D-dimer, functional fibrinogen, INR, prothrombin time, partial thromboplastin time (PTT), alanine aminotransferase, aspartate aminotransferase (AST), total bilirubin, creatinine, blood urea nitrogen (BUN), C-reactive protein, and bicarbonate; (5) comorbidities determined by *International Classification of Diseases, Ninth Revision* and *International Classification of Diseases, Tenth Revision* diagnostic codes: pneumonia, stroke, chronic kidney disease, type 2 diabetes, heart failure, myocardial infarction, and coronary heart disease; (6) interventions: mechanical ventilation, continuous renal replacement therapy, fluid intake, vasopressors, glucocorticoids, and antibiotics; and (7) outcomes: 28-day mortality after ICU admission and in-hospital mortality. For vital signs and laboratory tests, data within 24 hours prior to ICU admission were included. If multiple measurements were available, the mean value was used. The primary outcome of this study was 28-day mortality after ICU admission. Patients without a death record within 28 days of ICU admission were considered survivors.

### Feature Engineering

Feature engineering was performed in three steps:

First step was identification and handling of missing values. The R package “VIM” was used to identify missing values. To minimize bias caused by missing data, variables with more than 30% missing values were excluded. The percentage of missing values in the database is shown in Table S2 in Multimedia Appendix 1. Missing data imputation was performed using multiple imputation (MI) via the R package *mice*. Crucially, to prevent potential data leakage, imputation was conducted strictly after data splitting. The imputation models were trained exclusively on the training set and subsequently applied to the internal validation set. Furthermore, given the distinct clinical environments and potential differences in missingness mechanisms, the external validation datasets (the eICU-CRD and the Tianjin Medical University General Hospital cohort) were imputed independently. We evaluated 3 methods: predictive mean matching, classification and regression trees (CART), and random forest (RF). The CART algorithm was identified as the optimal method due to its superior convergence and distributional consistency. Standard MI typically requires pooling models via Rubin rules; however, this is computationally prohibitive and clinically impractical for complex, nonlinear ML algorithms like XGBoost. Therefore, as a widely adopted pragmatic approach in predictive modeling, the CART imputation was repeated 10 times to account for imputation uncertainty. The 10 resulting complete datasets were then aggregated into a single robust dataset by calculating the mean for continuous variables and the mode for categorical variables, which was subsequently used for model training and validation.

Second step was handling of outliers. Given the presence of equipment artifacts and data entry errors inherent in large electronic health records, a rigorous data cleaning procedure was implemented prior to analysis. We established clinically plausible physiological ranges for continuous variables based on clinical domain expertise. Values falling outside these physiologically possible ranges were defined as artifactual errors and were converted to missing values prior to imputation. However, extreme values that remained within biologically plausible limits were intentionally retained, as they reflect genuine severe pathological states in critically ill patients. For example, in the context of severe sepsis and systemic hypoperfusion, profound hyperlactatemia (eg, lactate >15 mmol/L) or severely elevated transaminases (eg, AST >1000 U/L) are true manifestations of severe metabolic derangement and ischemic hepatitis (shock liver), rather than measurement errors [21]. This strategy ensures data integrity while preserving valuable high-risk prognostic signals.

Third step was feature selection for model construction. The final feature set for the ML models was selected based on the training set using LASSO (least absolute shrinkage and selection operator) regression, the Boruta algorithm, RF-based recursive feature elimination with cross-validation (RFECV), and multivariable logistic regression. While simpler single-method strategies exist, clinical datasets are often noisy and highly collinear. Therefore, we adopted a multimethod ensemble approach to overcome the intrinsic biases of individual algorithms. Specifically, LASSO regression introduces L1 regularization into the objective function, selecting features and reducing dimensionality by shrinking coefficients, retaining features with significant contributions, and eliminating redundant ones [22]. The Boruta algorithm identifies the most important features by comparing the *Z*-value of each feature with that of the “shadow features” [23]. Recursive feature elimination (RFE), acting as a greedy algorithm, ranks and selects features based on their importance through iterative training, combined with 10-fold cross-validation to select clinical variables [24]. Furthermore, 10-fold cross-validation was embedded within both the LASSO and RFECV procedures, serving as an inherent feature-stability assessment. Prior to final confirmation, the variance inflation factor (VIF) was calculated [25]. A VIF of 1 indicates no multicollinearity; a VIF between 1 and 5 indicates moderate collinearity; a VIF >5 indicates high collinearity; and a VIF >10 indicates severe multicollinearity. Variables exhibiting high collinearity were accordingly removed. The features commonly selected across methods were finally evaluated using a multivariable logistic regression model to adjust for confounding effects and confirm them as statistically independent risk factors for the prognosis of SIC.

### Statistical Analysis

The Shapiro-Wilk test was used to assess the normality of continuous variables. Continuous variables conforming to a normal distribution were expressed as mean (SD), while those with a nonnormal distribution were expressed as median (IQR). Categorical variables were presented as counts and percentages. For comparisons of continuous variables between groups, a 2-tailed independent sample *t* test was used if the assumption of normality was met; otherwise, the Wilcoxon rank-sum test was applied. Pearson chi-square (*χ*²) test was used for categorical variables, while Fisher exact test was used when the expected frequency was <1 or the total sample size was <40. All statistical tests and calculations were performed using Python (version 3.11; Python Software Foundation) and the R package “tableone.” All charts were generated using the R package “ggplot2.” A *P* value <.05 was considered statistically significant.

### Model Development and Comparison

The MIMIC-IV database was randomly split into a training set (5586/7980, 70%) and an internal validation set (2394/7980, 30%). Ten features were used to construct the task-specific models. Stratified sampling was performed using the R package “createDataPartition” to ensure a balanced distribution of the original outcomes between the 2 subsets. A total of 12 ML algorithms were used to construct the models, including logistic regression, support vector machine, decision tree (DT), gradient boosting decision tree, light gradient boosting machine, categorical boosting, RF, naive Bayes, k-nearest neighbors, adaptive boosting, XGBoost, and artificial neural network. To comprehensively evaluate the models, we used a multidimensional assessment strategy. Initially, fundamental classification metrics, including AUC, sensitivity, specificity, accuracy, false negative rate (FNR), false positive rate (FPR), and *F*_1_-score, were calculated. Furthermore, considering the potential class imbalance, the precision-recall curve was adopted [26]. To assess clinical utility and reliability, calibration curves were used to evaluate the model’s calibration, and DCA was performed using the R package “rmda” to quantify the clinical net benefit [27].

To enhance the reliability and robustness of the models, both internal and external validations were performed. The internal validation set was derived from the MIMIC-IV database. Two independent datasets were used for external validation: the eICU-CRD database as external validation set I and the Tianjin Medical University General Hospital database as external validation set II. To address the class imbalance issue that could significantly distort performance metrics, the synthetic minority oversampling technique was applied during model training [28]. The SHAP algorithm was used to interpret the predictions of the ML models by calculating the SHAP value for each feature, quantifying their contributions to the prediction outcomes, and identifying critical features that influence model decisions [29]. Subsequently, univariate and multivariable logistic regression and regression-based prediction models were used to compare the ML model with 4 commonly used clinical risk assessment models (SOFA, APACHE II, SAPS II, and OASIS) in the internal validation set and in external validation set I. External validation set II served as an independent dataset that more closely resembled real-world clinical scenarios and was used to further validate the clinical applicability of the optimal model. Furthermore, to assess internal consistency and mitigate the risk of overfitting, 10-fold cross-validation was applied during the training phase for the prognostic models.

## Results

### Baseline Characteristics

**Figure 1.**
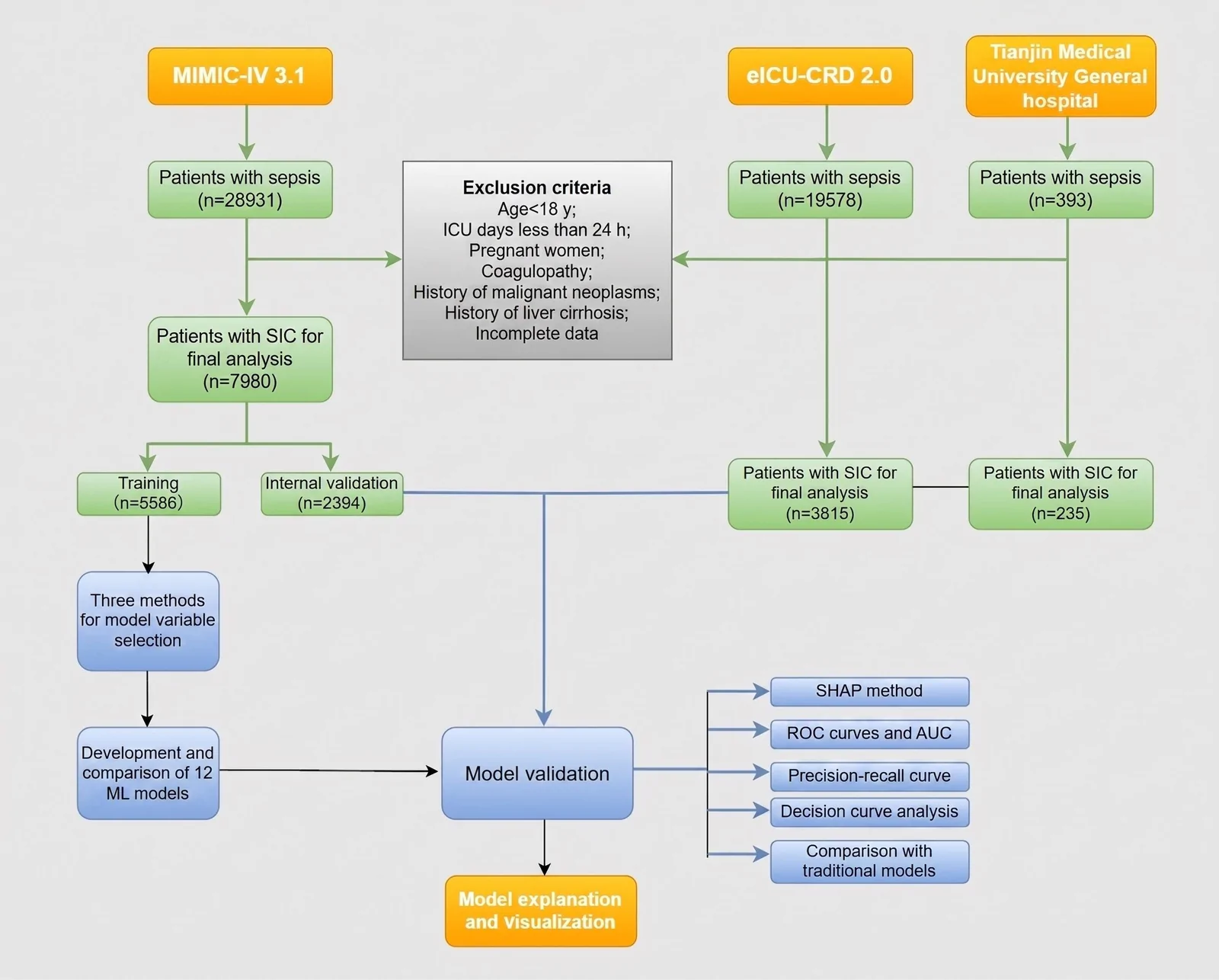
Flowchart of the study design. AUC: area under the curve; eICU-CRD: eICU Collaborative Research Database; ICU: intensive care unit; MIMIC-IV: Medical Information Mart for Intensive Care IV; ML: machine learning; ROC: receiver operating characteristic; SHAP: Shapley additive explanations; SIC: sepsis-induced coagulopathy.

As shown in Figure 1, from an initial cohort of 28,931 patients with sepsis in the MIMIC-IV database, 20,951 (72.4%) were excluded and 7980 (27.6%) were included; of 19,578 patients with sepsis in the eICU-CRD database, 15,763 (80.5%) were excluded and 3815 (19.5%) were included; and of 393 patients with sepsis from Tianjin Medical University General Hospital, 158 (40.2%) were excluded and 235 (59.8%) were included. In total, 12,030 patients with SIC were analyzed across the 3 cohorts, comprising 7980 (66.3%), 3815 (31.7%), and 235 (2.0%) patients, respectively. Table 1 summarizes the baseline demographic and clinical characteristics of the MIMIC-IV cohort, with a focus on 28-day mortality as the primary outcome and in-hospital mortality as the secondary outcome. Among these participants, 6015 survived (75.4%) and 1965 died (24.6%). The overall extent of missing data is presented in Table S2 in Multimedia Appendix 1. Clinical data for the 2 external validation cohorts are provided in Tables S3 and S4 in Multimedia Appendix 1.

**Table 1.** Baseline demographic and clinical characteristics of the Medical Information Mart for Intensive Care IV cohort.

Variables	Overall (N=7980)	Survival (n=6015)	Death (n=1965)	*P* value
Demographic characteristics, mean (SD)				
Age (y)	66.54 (16.25)	65.89 (16.49)	68.53 (15.31)	<.001
Weight (kg)	83.51 (24.71)	83.67 (24.71)	83.04 (24.69)	.33
Gender, n (%)				.03
Women	3160 (39.6)	2340 (38.9)	820 (41.7)	
Men	4820 (60.4)	3675 (61.1)	1145 (58.3)	
Vital signs, mean (SD)				
Heart rate (beats/min)	89.77 (17.35)	88.61 (16.95)	93.33 (18.07)	<.001
SBP^a^ (mm Hg)	111.96 (21.45)	113.12 (20.93)	108.42 (15.52)	<.001
DBP^b^ (mm Hg)	64.04 (14.19)	64.71 (14.55)	61.99 (12.96)	.20
Respiratory rate (breaths/min)	21.05 (5.21)	20.91 (5.04)	21.51 (5.35)	.34
SpO_2_^c^ (%)	96.88 (3.12)	97.15 (2.98)	96.05 (3.46)	.25
Temperature (°C)	36.78 (0.63)	36.81 (0.59)	36.73 (0.68)	.3
Laboratory tests, mean (SD)				
Hemoglobin (g/dL)	9.96 (1.98)	10.03 (1.94)	9.75 (2.08)	<.001
Platelet count (×10^9^/L)	148.85 (68.66)	150.10 (67.56)	145.01 (71.92)	.08
RDW^d^ (%)	16.26 (2.74)	15.94 (2.56)	17.24 (3.03)	<.001
WBC^e^ (×10^9^/L)	13.45 (6.84)	12.93 (5.78)	15.06 (9.54)	<.001
Anion gap (mmol/L)	15.23 (4.67)	14.51 (4.05)	17.43 (5.65)	<.001
Calcium (mmol/L)	8.13 (0.80)	8.12 (0.77)	8.17 (0.89)	.03
Glucose (mg/dL)	142.59 (57.89)	140.06 (51.61)	150.36 (73.35)	<.001
Potassium (mmol/L)	4.21 (0.63)	4.17 (0.60)	4.34 (0.72)	<.001
Sodium (mmol/L)	137.84 (5.59)	137.89 (5.28)	137.67 (6.45)	.17
Lactate (mmol/L)	2.71 (2.22)	2.36 (1.61)	3.78 (3.25)	<.001
INR^f^	2.02 (1.10)	1.94 (1.05)	2.26 (1.20)	<.001
PT^g^ (s)	21.74 (11.21)	20.88 (10.59)	24.36 (12.57)	<.001
PTT^h^ (s)	42.45 (19.75)	40.71 (18.28)	47.78 (22.89)	<.001
ALT^i^ (U/L)	145.05 (204.12)	142.41 (200.32)	153.21 (215.92)	.08
AST^j^ (U/L)	210.55 (345.60)	195.80 (315.40)	255.70 (420.85)	<.001
Total bilirubin (mg/dL)	3.44 (6.29)	2.90 (5.26)	5.08 (8.49)	<.001
Creatinine (mg/dL)	1.79 (1.61)	1.69 (1.60)	2.12 (1.57)	<.001
BUN^k^ (mg/dL)	34.77 (26.18)	31.94 (24.45)	43.44 (29.23)	<.001
Bicarbonate (mmol/L)	21.57 (4.69)	22.03 (4.41)	20.15 (5.21)	<.001
Comorbidities, n (%)				
Pneumonia				<.001
No	5208 (65.3)	4102 (68.2)	1106 (56.3)	
Yes	2772 (34.7)	1913 (31.8)	859 (43.7)	
Stroke				.01
No	7112 (89.1)	5391 (89.6)	1721 (87.6)	
Yes	868 (10.9)	624 (10.4)	244 (12.4)	
CKD^l^				.005
No	6251 (78.3)	4756 (79.1)	1495 (76.1)	
Yes	1729 (21.7)	1259 (20.9)	470 (23.9)	
Diabetes				.19
No	5665 (71.0)	4247 (70.6)	1418 (72.2)	
Yes	2315 (29.0)	1768 (29.4)	547 (27.8)	
Heart failure				.08
No	5348 (67.0)	4063 (67.6)	1285 (65.4)	
Yes	2632 (33.0)	1952 (32.4)	680 (34.6)	
MI^m^				<.001
No	7369 (92.3)	5625 (93.5)	1744 (88.8)	
Yes	611 (7.67)	390 (6.5)	221 (11.2)	
CHD^n^				.85
No	5318 (66.6)	4005 (66.6)	1313 (66.8)	
Yes	2662 (33.4)	2010 (33.4)	652 (33.2)	
Treatment measures, n (%)				
CRRT^o^				<.001
No	7044 (88.3)	5560 (92.4)	1484 (75.5)	
Yes	936 (11.7)	455 (7.6)	481 (24.5)	
Ventilation				<.001
No	1152 (14.4)	929 (15.4)	223 (11.4)	
Yes	6828 (85.6)	5086 (84.6)	1742 (88.6)	
Vasopressor				<.001
No	2736 (34.3)	2415 (40.2)	321 (16.3)	
Yes	5244 (65.7)	3600 (59.8)	1644 (83.7)	
GC^p^				<.001
No	5370 (67.3)	4198 (69.8)	1172 (59.6)	
Yes	2610 (32.7)	1817 (30.2)	793 (40.4)	
Antibiotic				<.001
No	111 (1.4)	111 (1.9)	0.00 (0.0)	
Yes	7869 (98.6)	5904 (98.1)	1965.00 (100.0)	
Input amount (mL), mean (SD)	5188.75 (3923.14)	5137.31 (3769.94)	5346.20 (4356.02)	.06
SIC^q^ score				<.001
4	5013 (62.8)	3959 (65.8)	1054 (53.6)	
5	1757 (22.0)	1303 (21.7)	454 (23.1)	
6	1210 (15.2)	753 (12.5)	457 (23.3)	
Clinical risk assessment models, mean (SD)				
SAPS II^r^	43.54 (15.10)	40.42 (13.47)	53.11 (15.80)	<.001
OASIS^s^	34.53 (8.87)	33.04 (8.25)	39.09 (9.13)	<.001
SOFA^t^	7.64 (3.86)	6.95 (3.41)	9.78 (4.34)	<.001
APACHE II^u^	21.49 (7.45)	20.09 (6.81)	25.77 (7.69)	<.001
Outcome, mean (SD)				
ICU^v^ days	5.75 (6.96)	5.69 (7.47)	5.96 (5.11)	.07
Hospital death, n (%)				<.001
No	7334 (91.9)	5759 (95.7)	1575 (80.2)	
Yes	646 (8.1)	256 (4.3)	390 (19.8)	

a SBP: systolic blood pressure.

b DBP: diastolic blood pressure.

c SpO_2_: oxygen saturation.

d RDW: red blood cell distribution width.

e WBC: white blood cells.

f INR: international normalized ratio.

g PT: prothrombin time.

h PTT: partial thromboplastin time.

i ALT: alanine aminotransferase.

j AST: aspartate aminotransferase.

k BUN: blood urea nitrogen.

l CKD: chronic kidney disease.

m MI: myocardial infarction.

n CHD: coronary heart disease.

o CRRT: continuous renal replacement therapy.

p GC: glucocorticoids.

q SIC: sepsis-induced coagulopathy.

r SAPS II: Simplified Acute Physiology Score II.

s OASIS: Oxford Acute Severity of Illness Score.

t SOFA: Sequential Organ Failure Assessment.

u APACHE II: Acute Physiology and Chronic Health Evaluation II.

v ICU: intensive care unit.

The optimal ML algorithm was identified as “cart,” selected based on the convergence and consistency of the distribution between imputed and observed values. This process was repeated 10 times, and the mean of the results was used to impute missing data for further analysis.

### Variable Selection

**Figure 2.**
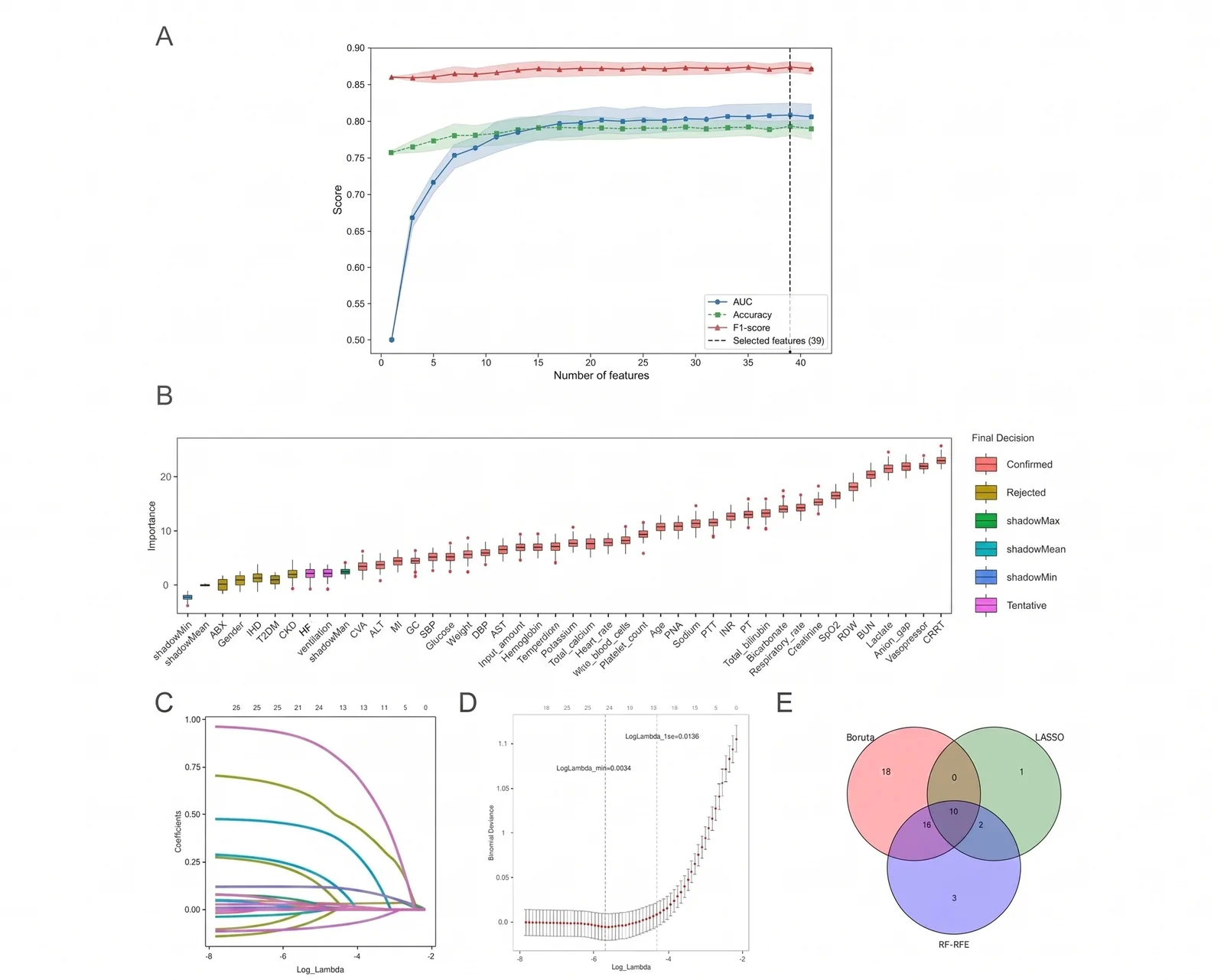
Identification and selection of features for model construction. (A) Variable selection procedure using the recursive feature elimination with cross-validation curve. (B) Feature importance ranking by the Boruta algorithm. (C) Least absolute shrinkage and selection operator (LASSO) coefficient profiles against log(lambda). (D) Selection of the optimal lambda in the LASSO model using cross-validation. (E) Venn diagram showing the intersection of features selected by the 3 methods. ABX: antibiotics; ALT: alanine aminotransferase; AST: aspartate aminotransferase; AUC: area under the curve; BUN: blood urea nitrogen; CKD: chronic kidney disease; CRRT: continuous renal replacement therapy; CVA: cerebrovascular accident; DBP: diastolic blood pressure; GC: glucocorticoids; HF: heart failure; IHD: ischemic heart disease; INR: international normalized ratio; MI: myocardial infarction; PNA: pneumonia; PT: prothrombin time; PTT: partial thromboplastin time; RDW: red blood cell distribution width; RF-RFE: random forest–based recursive feature elimination; SBP: systolic blood pressure; SpO_2_: oxygen saturation; T2DM: type 2 diabetes mellitus.

The initial selection of these 41 candidate variables was comprehensively determined based on a literature review of previous prognostic studies on sepsis and coagulopathy, established clinical guidelines, and the routine availability of these variables in the ICU. Specifically, demographic characteristics, vital signs, interventions, and laboratory parameters were included as they have been widely validated as crucial predictors of mortality in patients with sepsis [30-32]. The RFE method combined with 10-fold cross-validation was used for preliminary screening, retaining 39 variables associated with the risk of short-term mortality (Figure 2A). The Boruta algorithm selected 34 variables classified as “Confirmed”; the results of the Boruta-based feature selection are illustrated in Figure 2B. Subsequently, LASSO coefficient analysis was performed. By minimizing binomial deviance through 10-fold cross-validation, the optimal lambda value (lambda.1se=0.0136) was determined, identifying 19 variables with nonzero coefficients (Figure 2C). Figure 2D shows the cross-validation curve for LASSO regression, demonstrating the binomial deviance across different lambda values. The feature subset was determined by the intersection of the 3 selection methods (Figure 2E). Finally, multivariable logistic regression analysis identified 10 variables significantly associated with the outcome, including AG, RDW, lactate, total bilirubin, age, BUN, SpO_2_, PTT, heart rate, and vasopressors (Table S5 in Multimedia Appendix 1). All variables remained independently predictive after adjustment. VIF analysis showed no significant multicollinearity among the variables (Table S6 in Multimedia Appendix 1).

### Model Development and Validation

**Figure 3.**
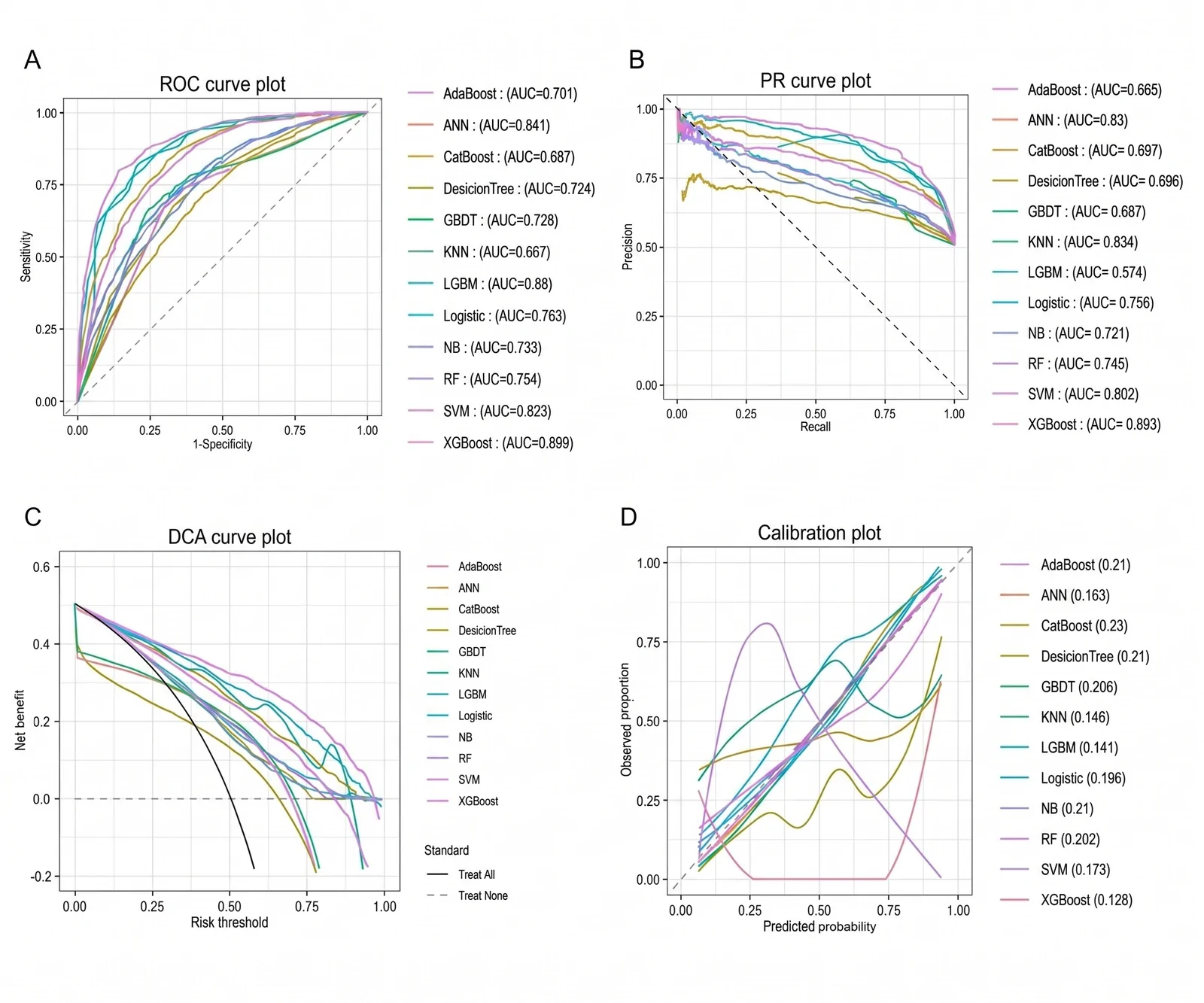
Performance evaluation of 12 machine learning models in the internal validation set. (A) Receiver operating characteristic (ROC) curves. (B) Precision-recall (PR) curves. (C) Decision curve analysis (DCA). (D) Calibration plots comparing predicted probabilities with observed outcomes. AdaBoost: adaptive boosting; ANN: artificial neural network; AUC: area under the curve; CatBoost: categorical boosting; GBDT: gradient boosting decision tree; KNN: k-nearest neighbors; LGBM: light gradient boosting machine; NB: naive Bayes; RF: random forest; SVM: support vector machine; XGBoost: extreme gradient boosting.

**Figure 4.**
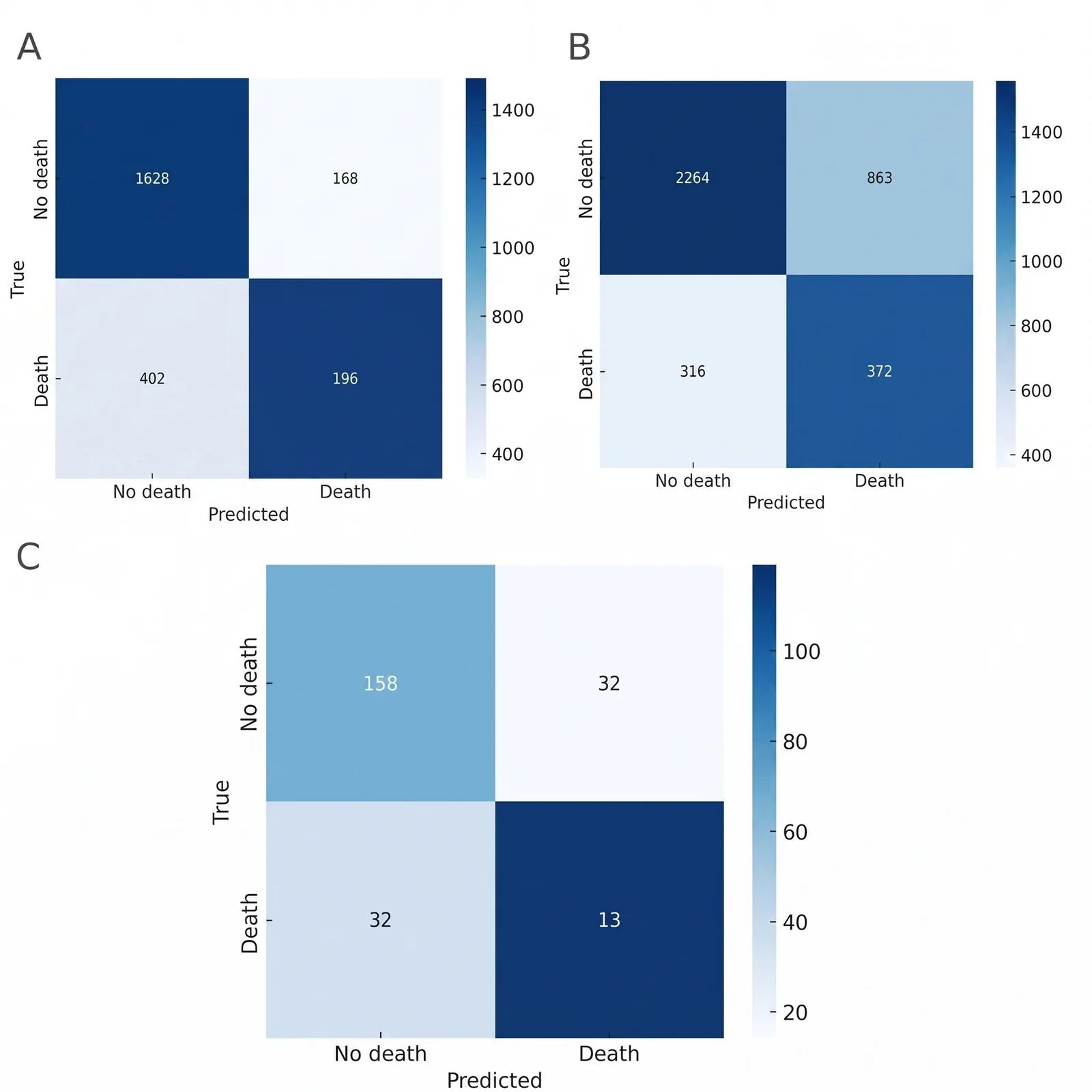
Confusion matrices for predicting 28-day mortality across different cohorts. (A) Internal validation set. (B) External validation set I. (C) External validation set II. The x-axis represents the predicted outcome, and the y-axis represents the actual outcome. The diagonal elements represent correct predictions (true negatives and true positives), while the off-diagonal elements represent misclassifications. Darker colors indicate a higher number of patients.

Based on the identified features, 12 ML models were developed to predict the prognosis of patients with SIC. The AUC, accuracy, recall, *F*_1_-score, specificity, FPR, and FNR were calculated to comprehensively evaluate the models. Based on these evaluations, the XGBoost model demonstrated the best performance. In the internal validation cohort, the XGBoost model achieved an area under the receiver operating characteristic curve (AUROC) of 0.899 (95% CI 0.889‐0.909), with accuracy, *F*_1_-score, and specificity of 0.818, 0.819, and 0.817, respectively (Table 2 and Figure 3). The confusion matrix revealed 1628 true negatives (TNs), 196 true positives (TPs), 402 false negatives (FNs), and 168 false positives (FPs) regarding 28-day mortality (Figure 4A). The model’s performance was further validated using external validation sets I and II. In external validation set I, the AUROC was 0.882 (95% CI 0.867‐0.897), and the model’s accuracy, *F*_1_-score, and specificity were 0.805, 0.803, and 0.813, respectively (Table S7 in Multimedia Appendix 1). The confusion matrix showed 2264 TNs, 372 TPs, 316 FNs, and 863 FPs (Figure 4B). External validation set II assessed the clinical applicability of the model, with an AUROC of 0.904 (95% CI 0.846‐0.962), with accuracy, *F*_1_-score, and specificity of 0.815, 0.811, and 0.818, respectively (Table S7 in Multimedia Appendix 1). The confusion matrix indicated 158 TN, 13 TP, 29 FN, and 30 FP (Figure 4C). Finally, the 12 models were used to evaluate in-hospital mortality in patients with SIC within the internal validation cohort. The results showed that the XGBoost model continued to perform excellently, with an AUROC (95% CI) of 0.963 (0.958‐0.968; Table S8 in Multimedia Appendix 1), further confirming the model’s predictive accuracy for the prognosis of SIC.

**Table 2.** Evaluation of each machine learning algorithm.

Model	Accuracy^a^	Recall^b^	*F*_1_-score^c^	AUROC^d^	Specificity^e^	FNR^f^	FPR^g^
XGBoost^h^	0.818	0.819	0.819	0.899	0.817	0.181	0.183
KNN^i^	0.769	0.895	0.797	0.867	0.642	0.105	0.358
ANN^j^	0.746	0.751	0.749	0.823	0.742	0.249	0.258
SVM^k^	0.659	0.612	0.644	0.733	0.706	0.388	0.294
CatBoost^l^	0.756	0.774	0.762	0.841	0.737	0.226	0.263
NB^m^	0.628	0.552	0.599	0.687	0.705	0.448	0.295
RF^n^	0.683	0.707	0.692	0.754	0.658	0.293	0.342
Logistic regression	0.692	0.661	0.684	0.762	0.724	0.339	0.276
LGBM^o^	0.790	0.802	0.794	0.880	0.777	0.198	0.223
AdaBoost^p^	0.701	0.718	0.708	0.701	0.683	0.282	0.317
GBDT^q^	0.705	0.719	0.711	0.725	0.691	0.281	0.309
Decision tree	0.668	0.794	0.707	0.724	0.540	0.206	0.460

a Accuracy: (TP+TN)/(P+N) represents the overall classification correctness, where N=negative, P=positive, TN=true negative, and TP=true positive.

b Recall (sensitivity): TP/(TP+FN) represents the true positive rate, where FN=false negative and TP=true positive.

c *F*_1_-score: 2×(Precision×Recall)/(Precision+Recall) represents the harmonic mean.

d AUROC: area under the receiver operating characteristic curve.

e Specificity: TN/(TN+FP) represents the true negative rate, where FP=false positive and TN=true negative.

f FNR: false negative rate (FN/[FN+TP]).

g FPR: false positive rate (FP/[FP+TN]).

h XGBoost: extreme gradient boosting.

i KNN: k-nearest neighbors.

j ANN: artificial neural network.

k SVM: support vector machine.

l CatBoost: categorical boosting.

m NB: naive Bayes.

n RF: random forest.

o LGBM: light gradient boosting machine.

p AdaBoost: adaptive boosting.

q GBDT: gradient boosting decision tree.

Furthermore, to assess the robustness and generalizability of the XGBoost model, 10-fold cross-validation was performed to examine its predictive efficacy. Detailed performance metrics and ROC curve results (Table S9 and Figure S1 in Multimedia Appendix 1) indicated that the constructed model exhibited favorable robustness and generalizability, with no signs of overfitting or underfitting.

**Figure 5.**
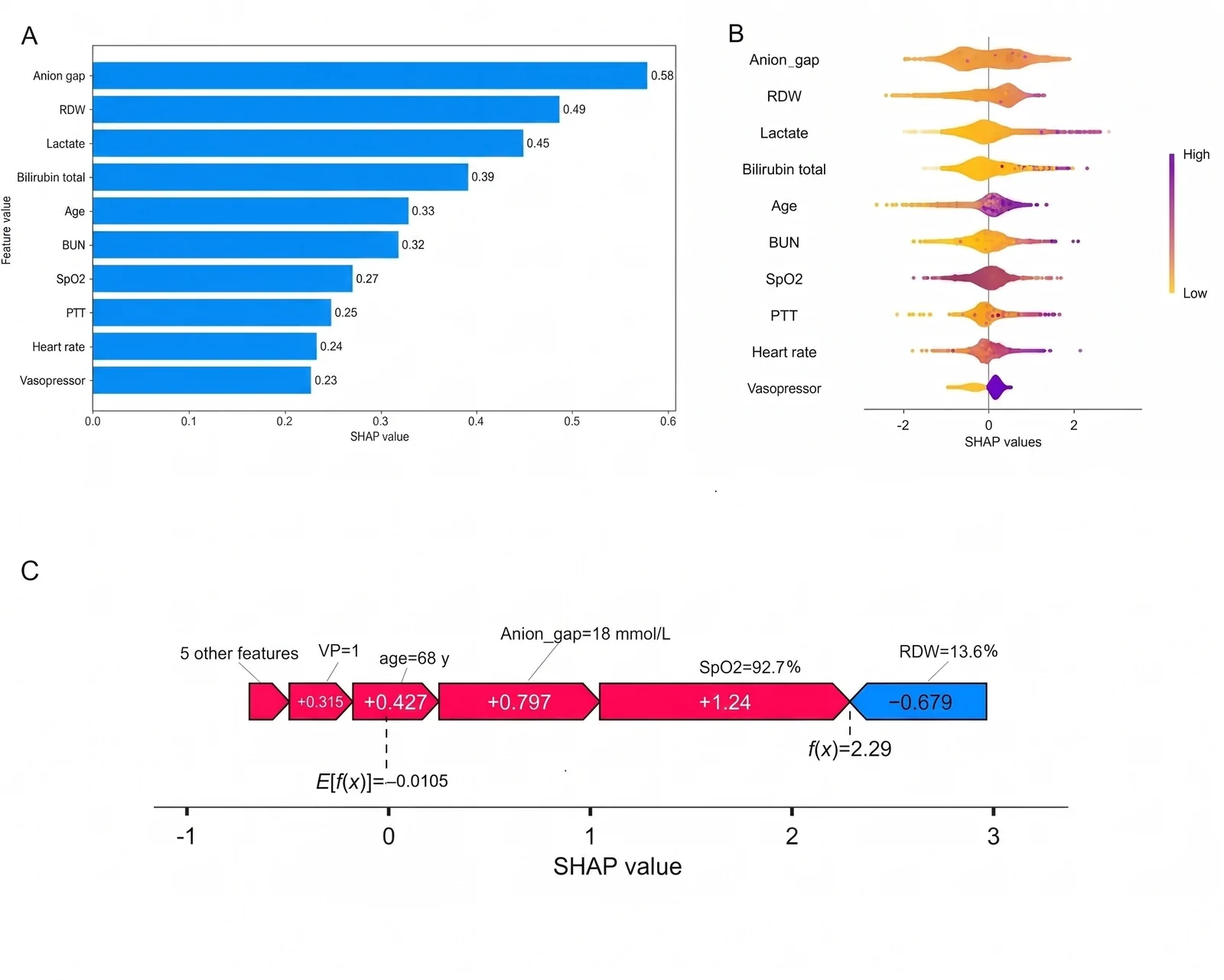
Visualization of feature importance and model interpretation using Shapley additive explanations (SHAP). (A) Bar plot illustrating the mean absolute SHAP values, ranking the top 10 features by their global importance in the XGBoost model. (B) SHAP summary plot. Each dot represents a single patient sample. The position on the x-axis indicates the SHAP value (impact on model output), while the color gradient represents the feature value (purple color indicates high values, and yellow color indicates low values). Positive SHAP values suggest an increased risk of mortality. (C) SHAP force plot for an individual patient case. Red arrows represent features that increase the prediction score (higher risk), while blue arrows represent features that decrease the score (lower risk). The length of the arrow reflects the magnitude of the feature’s contribution. BUN: blood urea nitrogen; PTT: partial thromboplastin time; RDW: red blood cell distribution width; SpO_2_: oxygen saturation; vp: vasopressor.

To enhance the interpretability of the model, the SHAP method was used to quantify the contribution of each input feature to the model’s predictions. The importance scores of the 10 features used in the XGBoost model were calculated to identify critical factors (Figure 5A and B). The position on the y-axis implicitly indicates the ranking of feature importance, while the x-axis represents the relationship between feature values and their corresponding SHAP values. For instance, the SHAP values for advanced age were generally greater than zero, indicating that the risk of mortality in patients with SIC increases with age. Furthermore, feature importance results based on absolute SHAP values revealed that the top 5 risk features for SIC were AG, RDW, lactate, total bilirubin, and age. Specifically, the analysis revealed a consistent positive correlation for the top 5 key predictors: higher levels of AG, elevated RDW, increased lactate concentrations, higher total bilirubin, and advanced age were all robustly associated with an increased predicted risk of 28-day mortality. For this specific patient, SHAP results indicated that the critical threshold values for predicting the probability of poor prognosis were AG=18 mmol/L, RDW=13.6%, SpO_2_=92.7%, and age=68 years (Figure 5C).

### Model Comparison

**Figure 6.**
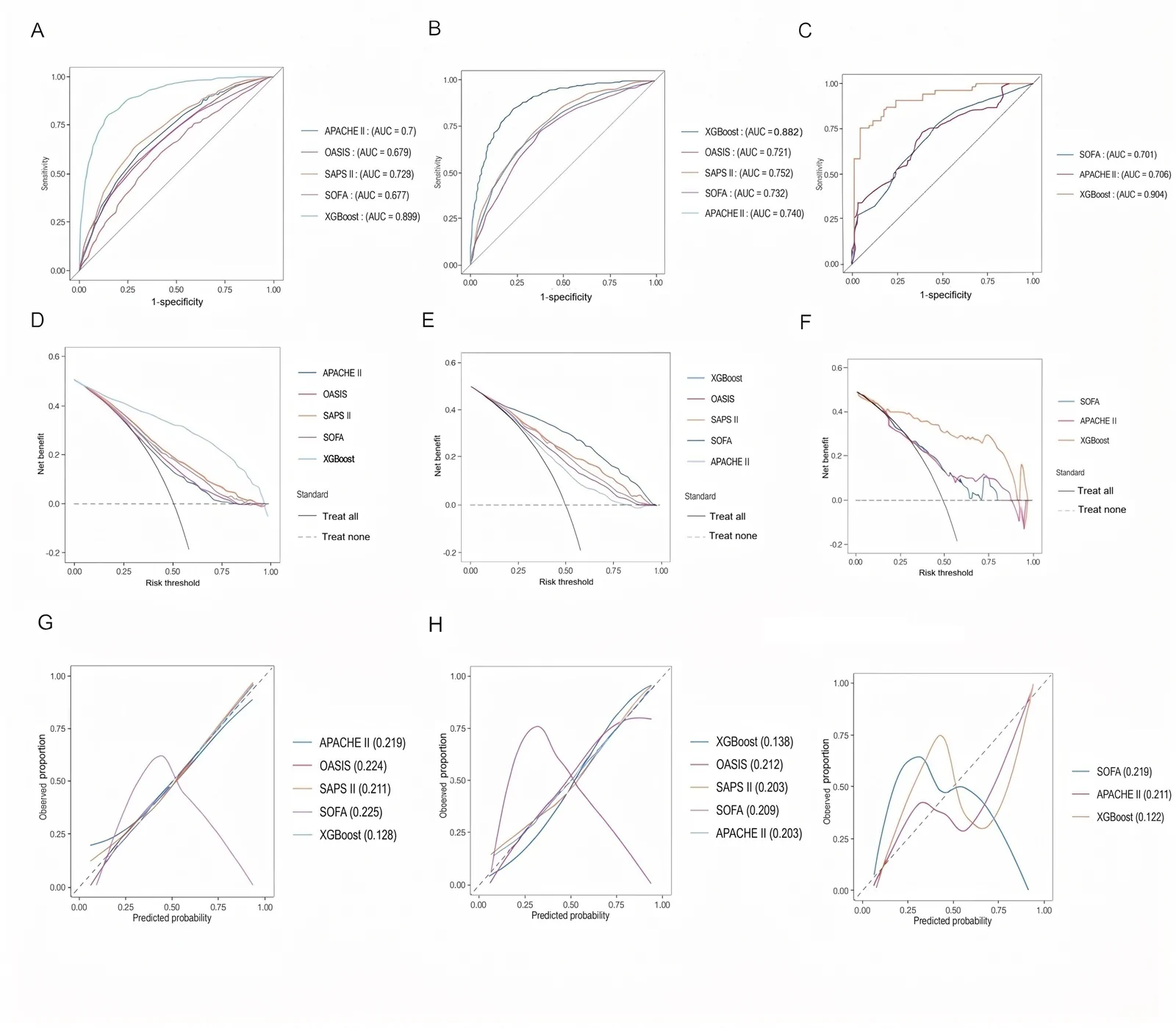
Performance comparison and clinical utility assessment of the XGBoost model vs traditional clinical risk assessment models. (A-C) Receiver operating characteristic curves comparing the discriminative ability of the XGBoost model with APACHE II, OASIS, SAPS II, and SOFA scores. (A) Internal validation set. (B) External validation set I. (C) External validation set II. (D-F) Decision curve analyses illustrating the clinical net benefit at different threshold probabilities. (D) Internal validation set. (E) External validation set I. (F) External validation set II. The black line represents the strategy of treating all patients, while the horizontal dashed line represents treating none. (G-I) Calibration plots assessing the agreement between predicted probabilities and observed outcomes. (G) Internal validation set. (H) External validation set I. (I) External validation set II. The diagonal dotted line represents ideal calibration. APACHE II: Acute Physiology and Chronic Health Evaluation II; AUC: area under the curve; OASIS: Oxford Acute Severity of Illness Score; SAPS II: Simplified Acute Physiology Score II; SOFA: Sequential Organ Failure Assessment; XGBoost: extreme gradient boosting.

The performance of the XGBoost model was compared with that of 4 clinical risk assessment models using the internal validation set. The XGBoost model demonstrated the best discriminative ability (AUC=0.899) (Figure 6A). Univariate logistic regression analysis revealed statistically significant odds ratios (ORs) for all models, with the XGBoost model exhibiting the largest effect size (OR 47.96, 95% CI 37.43‐61.81; *P*<.001). Multivariable regression analysis indicated significant predictive associations for the adjusted XGBoost model (OR 31.45, 95% CI 24.05‐41.35; *P*<.001), SAPS II, and OASIS, with the XGBoost model maintaining the strongest prognostic relationship (Table S10 in Multimedia Appendix 1).

The same analysis was repeated using external validation set I, and the XGBoost model consistently demonstrated the best predictive accuracy for 28-day mortality (AUC=0.882; Figure 6B). Univariate logistic regression analysis indicated that all models were statistically significant, with the XGBoost model retaining the most significant risk association (OR 77.47, 95% CI 54.98‐110.57; *P*<.001). Following multivariable adjustment, the XGBoost model (OR 52.05, 95% CI 36.23‐75.70; *P*<.001), SAPS II, OASIS, and APACHE II maintained significant prognostic associations, further confirming the robust predictive capability of the XGBoost model (Table S11 in Multimedia Appendix 1). Analysis of the external validation set II further assessed the model’s robust clinical adaptability (AUC=0.904; Figure 6C). Consistent with previous findings, logistic regression in this cohort revealed that the XGBoost model maintained an exceptionally strong association with mortality in both univariate (OR 122.34, 95% CI 30.21‐649.17; *P*<.001) and multivariable analyses (OR 113.88, 95% CI 24.32‐762.29; *P*<.001). In contrast, traditional scores showed limited predictive value in multivariable analysis (Table S12 in Multimedia Appendix 1).

### Clinical Utility

To objectively evaluate the clinical utility of the ML models in predicting 28-day mortality, calibration plots were generated using the bootstrap method, and DCA was performed for each model in the internal validation set and in 2 external validation sets. The DCA results indicated the net benefit of model-guided interventions across various threshold probabilities. A high net benefit indicates that using the model to trigger aggressive therapies, such as intensive fluid resuscitation, improves overall patient outcomes compared to treating all patients or none (Figure 6D-F). Calibration curve analysis demonstrated that the XGBoost model maintained high consistency between predicted probabilities and actual outcomes. This accuracy builds physician trust for individual risk stratification (Figure 6G-I). Considering the potential application of our model in future clinical practice, a user-friendly web-based tool, SIC Predict Streamlit, was developed [33]. This tool enables medical professionals to easily input key patient parameters to rapidly assess the mortality risk associated with SIC, thereby improving clinical resource utilization.

## Discussion

### Principal Findings

In this study, we developed and validated SIC clinical prognosis prediction models based on 12 ML algorithms using 3 independent databases. The performance of each model was evaluated using AUROC, accuracy, recall, *F*_1_-score, and specificity, identifying the XGBoost model as having the optimal performance. This model exhibited excellent discriminative ability and robustness in both internal and external validation sets, significantly outperforming traditional risk assessment tools (SOFA, APACHE II, SAPS II, and OASIS). Clinically, by translating these robust algorithms into an accessible online calculator, this study provides a highly interpretable decision support tool that enables frontline clinicians to achieve early risk stratification, guide timely individualized interventions, and optimize the allocation of intensive care resources.

SIC is a common and fatal complication in patients with sepsis, characterized by a vicious cycle of microvascular thrombosis and organ perfusion failure. The occurrence and severity of SIC are independently associated with in-hospital mortality [4,34]. Although the ISTH criteria provide a framework for identifying SIC, existing clinical scoring systems have inherent limitations in dynamic and individualized prognostic assessment [35,36]. Most of these models are based on linear assumptions, making it difficult to capture the highly nonlinear processes of physiological compensation and decompensation in SIC. In contrast, advanced ML algorithms, particularly tree-based ensemble methods such as XGBoost, are inherently designed to overcome these analytical bottlenecks. Unlike simpler linear models (eg, logistic regression), XGBoost iteratively constructs multiple DTs to minimize prediction errors, allowing it to automatically capture complex, higher-order interactions among diverse clinical variables [37]. This makes XGBoost exceptionally well-suited for modeling the intricate, multisystem pathophysiological cascades of SIC, thereby offering a more precise and individualized prognostic evaluation.

To enhance the robustness and reliability of the model and eliminate irrelevant or highly collinear features, feature selection was performed using the following 3 methods: LASSO regression, the Boruta algorithm, and RFECV. This process identified 10 independent predictors associated with the prognosis of SIC. Furthermore, to enhance interpretability and facilitate clinical adoption, we implemented SHAP to visualize feature contributions and elucidate the decision-making process of the model [38]. SHAP importance analysis revealed that the top 5 key features were AG, RDW, lactate, total bilirubin, and age. These indicators reflect the body’s metabolic status, organ function, and physiological reserve capacity and are closely correlated with the pathophysiological mechanisms of SIC.

AG is a crucial indicator for assessing acid-base balance and is commonly associated with metabolic acidosis [39]. Previous studies have shown that for each 1 mmol/L increase in AG, the risk of mortality can increase by 5% to 10% [40]. In patients with SIC, elevated AG suggests severe tissue hypoperfusion, cellular metabolic dysfunction, and multiple organ failure, serving as a comprehensive marker of disease severity. These represent the core pathological processes of sepsis-induced coagulation disorders. Sepsis leads to microcirculatory dysfunction, increasing the accumulation of acidic metabolites such as lactate, thereby widening the AG. A high AG not only indicates metabolic acidosis but is also associated with inflammatory cascades and coagulation activation, which further exacerbate endothelial injury and thrombosis, leading to a worse prognosis.

RDW reflects the heterogeneity of red blood cell size; its elevation is closely related to inflammatory responses, oxidative stress, and malnutrition [41]. In SIC, a persistent inflammatory state suppresses bone marrow function and shortens red blood cell lifespan, leading to increased RDW. This may worsen prognosis by affecting microcirculation and exacerbating coagulation abnormalities (such as platelet activation and fibrin deposition), reflecting the chronicity and systemic inflammatory burden of sepsis [42-44].

Lactate is a direct marker of tissue hypoxia and anaerobic metabolism [45]. Microcirculatory dysfunction and tissue hypoperfusion lead to increased lactate production and decreased clearance. Hyperlactatemia not only suggests circulatory failure but is also associated with the progression of coagulation dysfunction, including endothelial cell activation and thrombosis, ultimately leading to organ failure [46]. Sepsis-induced liver injury (such as ischemic hepatitis or inflammatory cytokine storms) causes bilirubin metabolism disorders, further aggravating coagulation abnormalities. This correlates with multiple organ failure, forming a vicious cycle [47].

Advanced age reduces physiological reserve and enhances a prothrombotic state; older patients are more prone to coagulation activation and endothelial dysfunction, resulting in worsened prognosis [48]. For older patients with SIC, clinical decision-making should be more prudent, necessitating a balance between treatment benefits and risks to formulate individualized treatment plans.

Beyond physiological interpretation, individualized SHAP force plots provide actionable bedside insights. Traditional models merely output a mortality probability. In contrast, SHAP explicitly identifies patient-specific pathophysiological drivers. For example, a high SHAP value for lactate directly alerts physicians to severe tissue hypoxia. This insight promptly guides targeted resuscitation strategies, including fluid optimization and vasopressor adjustments. Ultimately, SHAP transforms static predictions into dynamic guides for personalized critical care.

By integrating large-scale multicenter databases, this study compared the XGBoost model with various ML algorithms and traditional clinical risk prediction models (including SOFA, APACHE II, SAPS II, and OASIS). Both internal and external validation cohorts confirmed the superior predictive capability of the XGBoost model for early mortality. DCA and calibration curves further validated the model’s clinical utility and predictive calibration. Compared with other models, the XGBoost model exhibited the widest range of clinically reasonable risk-threshold probabilities and the highest net benefit. Traditional scoring systems are static, weight-based additive systems, whereas ensemble learning models like XGBoost can iteratively learn higher-order interactions among complex features through boosting techniques. For instance, the model may capture synergistic effects between advanced age and delayed lactate clearance or between high bilirubin levels and coagulation disorders, which are nonlinear relationships that traditional statistical models struggle to represent effectively. Furthermore, we did not settle for merely providing a predictive tool with a high AUC; instead, we comprehensively screened for the optimal model using multiple performance metrics. Furthermore, multiple feature selection methods were used to ensure the robustness and biological plausibility of the selected variables. This approach effectively reduced model complexity and the risk of overfitting, ensuring that the model focused on the most biologically significant features. As a result, it provides an efficient and interpretable decision support tool for the prognostic evaluation of SIC.

However, this study has several potential limitations. First, as a retrospective cohort study, it may be subject to selection bias and information bias, and causal relationships cannot be established. Future prospective studies are required to further validate model performance. Second, the model’s predictions are based on data obtained within the first 24 hours of ICU admission, without considering the dynamic evolution of the disease. Future research could develop dynamic prediction models incorporating time-series data to facilitate continuous risk assessment. Specifically, advanced deep learning architectures designed for sequential data hold great potential. Using models such as recurrent neural networks, long short-term memory networks, or transformers could enable the dynamic tracking of patient trajectories. Integrating these methodologies will facilitate continuous, real-time risk assessment and further improve prognostic accuracy. Third, although data from multiple centers were included, they were primarily from the United States and China, and the model’s performance in other ethnicities and regional populations remains unclear. Additionally, the relatively small sample size of external validation set II (n=235) may affect statistical power and the assessment of generalizability, necessitating further validation. Finally, due to data availability limitations, certain potentially important predictors (such as imaging features and genetic markers) were not included in the model, which may have restricted its predictive performance. Therefore, further validation using prospective, multicenter, and large-scale patient datasets will provide more robust evidence for the XGBoost model’s effectiveness and generalizability.

### Conclusions

This study developed and validated ML models based on large-scale multicenter databases for predicting early mortality in patients with SIC, demonstrating significant improvement over traditional clinical risk assessment tools across all metrics. SHAP analysis further confirmed the model’s interpretability. The findings provide a valuable reference for the prognostic assessment and individualized management of patients with SIC.

## Supplementary material

10.2196/90285 Multimedia Appendix 1 Sepsis-induced coagulopathy diagnostic criteria, missing data distributions, baseline patient characteristics, feature selection and collinearity diagnostics, and comprehensive performance metrics of predictive models across internal and external validation cohorts.

10.2196/90285 Checklist 1 TRIPOD+AI checklist.

## Abbreviations

AG: anion gap
APACHE II: Acute Physiology and Chronic Health Evaluation II
AST: aspartate aminotransferase
AUC: area under the curve
AUROC: area under the receiver operating characteristic curve
BUN: blood urea nitrogen
CART: classification and regression trees
DCA: decision curve analysis
DT: decision tree
EICU: emergency intensive care unit
eICU-CRD: eICU Collaborative Research Database
FN: false negative
FNR: false negative rate
FP: false positive
FPR: false positive rate
ICU: intensive care unit
INR: international normalized ratio
ISTH: International Society on Thrombosis and Haemostasis
LASSO: least absolute shrinkage and selection operator
MI: multiple imputation
MIMIC-IV: Medical Information Mart for Intensive Care IV
ML: machine learning
OASIS: Oxford Acute Severity of Illness Score
OR: odds ratio
PTT: partial thromboplastin time
RDW: red blood cell distribution width
RF: random forest
RFE: recursive feature elimination
RFECV: recursive feature elimination with cross-validation
SAPS II: Simplified Acute Physiology Score II
SHAP: Shapley additive explanations
SIC: sepsis-induced coagulopathy
SOFA: Sequential Organ Failure Assessment
SpO_2_: oxygen saturation
TN: true negative
TP: true positive
TRIPOD+AI: Transparent Reporting of a Multivariable Prediction Model for Individual Prognosis or Diagnosis for Artificial Intelligence
VIF: variance inflation factor
XGBoost: extreme gradient boosting
