# Supplementary material for: Development and Validation of an Interpretable Machine Learning Model to Predict Mortality in Patients With Sepsis-Induced Coagulopathy: Multicenter Cohort Study
Source: JMIR Med Inform. 2026 Sep 21;14:e90285. doi: 10.2196/90285 (PMC13592666; doi:10.2196/90285)
Supplement: Supplementary file 2 [file medinform-v14-e90285-s001.docx]

**Table S1.** Sepsis‐induced coagulopathy (SIC) criteria.

|  | **Score** | **Range** |
| --- | --- | --- |
| Platelet count (−10^9^/L) | 2 | < 100 |
|  | 1 | ≧ 100, < 150 |
| INR^a^ | 2 | > 1.4 |
|  | 1 | > 1.2, ≦1.4 |
| SOFA^b^ score | 2 | ≧ 2 |
|  | 1 | 1 |
| Total score for SIC^c^ |  | ≥4 |

SOFA score is the sum of 4 items (respiratory SOFA, cardiovascular SOFA, hepatic SOFA, renal SOFA). ^a^*INR: international normalized ratio, ^b^SOFA: sequential organ failure assessment, ^c^SIC: sepsis‐induced coagulopathy.*

**Table S2.** The percentage of missing values for variables in the MIMIC-IV database.

| **Variable Names** | **Overall** | **Missing** |
| --- | --- | --- |
| n | 7980 |  |
| Age | 68 (18-99) | 0.0 |
| Weight | 80 (1-570) | 1.0 |
| Height | 170 (122-203) | 38.4 |
| Absolute lymphocyte count | 0.86 (0-259.18) | 71.6 |
| Hemoglobin | 9.75 (3.7-19.35) | 0.1 |
| Platelet count | 118.55 (6-1200.5) | 0.0 |
| RDW | 15.67 (11.47-33.5) | 0.1 |
| White blood cells | 11.23 (0.1-386.7) | 0.1 |
| Absolute neutrophil count | 9.64 (0-117.83) | 71.6 |
| Albumin | 2.9 (0.9-5.35) | 49.3 |
| Anion gap | 14.5 (3-48.8) | 0.2 |
| Total calcium | 8.1 (4.66-17.23) | 5.0 |
| Glucose | 127.67 (34-886.33) | 0.4 |
| Potassium | 4.13 (2.2-7.6) | 0.1 |
| Total protein | 5.6 (2.3-14.7) | 97.2 |
| Sodium | 138 (105.33-175) | 0.1 |
| Lactate | 2.1 (0.3-23.89) | 16.5 |
| D-dimer | 3419 (223-21081) | 98.2 |
| Fibrinogen functional | 223.165 (26-1252) | 58.3 |
| INR | 1.7 (0.9-19.37) | 0.0 |
| PT | 18.25 (9.4-150) | 0.0 |
| PTT | 35.8 (18.5-150) | 1.2 |
| ALT | 38 (2-20243.33) | 25.9 |
| AST | 67 (3-34532.5) | 25.4 |
| Total bilirubin | 1.4 (0.1-73.5) | 25.4 |
| Creatinine | 1.25 (0.1-19.9) | 0.0 |
| BUN | 27 (1-213.33) | 0.0 |
| Creactive protein | 121 (1.3-531.3) | 96.4 |
| Bicarbonate | 21.67 (5-45) | 0.0 |
| Input amount | 4200.065 (30-34642.12) | 10.4 |
| SOFA | 7 (0-23) | 0.0 |
| APACHEII | 21 (3-54) | 0.0 |
| Heart rate | 88.63 (37.59-162.43) | 0.1 |
| SBP | 108.95 (38-5527.5) | 10.5 |
| DBP | 61.44 (19-11410.33) | 10.5 |
| MAP | 74.41 (-32-1227.75) | 59.2 |
| Respiratory rate | 19.61 (0-269266.77) | 0.2 |
| SpO_2_ | 96.97 (53.43-32539.96) | 0.1 |
| Temperature | 98.29 (0-247.2) | 8.4 |
| SAPSII | 42 (6-114) | 0.0 |
| OASIS | 34 (7-72) | 0.0 |
| Gender (%) |  |  |
| Female | 3160 (39.6) | 0.0 |
| Male | 4820 (60.4) |  |
| Shock (%) |  |  |
| No | 5543 (69.5) | 0.0 |
| Yes | 2437 (30.5) |  |
| DIC (%) |  |  |
| No | 7842 (98.3) | 0.0 |
| Yes | 138 (1.7) |  |
| AKI (%) |  |  |
| No | 3456 (43.3) | 0.0 |
| Yes | 4524 (56.7) |  |
| PNA (%) |  |  |
| No | 5208 (65.3) | 0.0 |
| Yes | 2772 (34.7) |  |
| CVA (%) |  |  |
| No | 7112 (89.12) | 0.0 |
| Yes | 868 (10.88) |  |
| CKD (%) |  |  |
| No | 6251 (78.3) | 0.0 |
| Yes | 1729 (21.7) |  |
| T2dm (%) |  |  |
| No | 5665 (71.0) | 0.0 |
| Yes | 2315 (29.0) |  |
| HF (%) |  |  |
| No | 5348 (67.0) | 0.0 |
| Yes | 2632 (33.0) |  |
| MI (%) |  |  |
| No | 7369 (92.3) | 0.0 |
| Yes | 611 (7.7) |  |
| IHD (%) |  |  |
| No | 5318 (66.6) | 0.0 |
| Yes | 2662 (33.4) |  |
| crrt (%) |  |  |
| No | 7044 (88.3) | 0.0 |
| Yes | 936 (11.7) |  |
| Ventilation (%) |  |  |
| No | 1152 (14.4) | 0.0 |
| Yes | 6828 (85.6) |  |
| Vasopressor (%) |  |  |
| No | 2736 (34.3) | 0.0 |
| Yes | 5244 (65.7) |  |
| GC (%) |  |  |
| No | 5370 (67.3) | 0.0 |
| Yes | 2610 (32.7) |  |
| ABX (%) |  |  |
| No | 111 (1.4) | 0.0 |
| Yes | 7869 (98.6) |  |
| SIC score (%) |  |  |
| 4 | 5013 (62.8) | 0.0 |
| 5 | 1757 (22.0) |  |
| 6 | 1210 (15.2) |  |
| ICU day | 3.38 (1.01-159.67) | 0.0 |
| Hosp dead (%) |  |  |
| No | 7334 (91.9) | 0.0 |
| Yes | 646 (8.1) |  |
| Death within 28days (%) |  |  |
| No | 6015 (75.4) | 0.0 |
| Yes | 1965 (24.6) |  |

**Table S3.** Baseline characteristics of SIC patients in eICU-CRD.

| **Variable** | **Overall** | **Survival** | **Death** | ***P* value** |
| --- | --- | --- | --- | --- |
|  | N = 3,815 | N = 3,127 | N = 688 |  |
| Age, year | 65.86 (15.5) | 65.73 (15.8) | 66.44 (14.5) | .26 |
| RDW, % | 16.74 (2.80) | 16.58 (2.74) | 17.49 (2.95) | <.001 |
| Anion gap, mmol/l | 13.32 (5.84) | 12.75 (5.35) | 15.90 (7.13) | <.001 |
| Lactate, mmol/L | 3.54 (3.58) | 2.96 (2.71) | 6.18 (5.41) | <.001 |
| PTT, seconds | 47.42 (25.80) | 46.13 (24.59) | 53.31 (30.03) | <.001 |
| BUN, mg/dL | 40.32 (27.43) | 38.83 (26.56) | 47.11 (30.17) | <.001 |
| Total bilirubin, mg/dL | 2.26 (3.65) | 2.03 (3.27) | 3.30 (4.86) | <.001 |
| Heart rate, times/minute | 113.49 (23.73) | 112.45 (23.42) | 118.23 (24.57) | <.001 |
| SpO_2_, % | 89.64 (9.84) | 90.56 (8.32) | 85.47 (14.17) | <.001 |
| Vasopressor, n (p%) |  |  |  | <.001 |
| No | 2,499 (65.5) | 2,153 (68.9) | 346 (50.3) |  |
| Yes | 1,316 (34.5) | 974 (31.2) | 342 (49.7) |  |
| SIC_score, n (p%) |  |  |  | <.001 |
| 4 | 2,238 (58.7) | 1,910 (61.1) | 328 (47.7) |  |
| 5 | 863 (22.6) | 705 (22.5) | 158 (23.0) |  |
| 6 | 714 (18.7) | 512 (16.4) | 202 (29.3) |  |
| SAPSII | 44.43 (16.45) | 41.60 (14.78) | 57.27 (17.49) | <.001 |
| OASIS | 32.24 (10.54) | 30.78 (9.91) | 38.87 (10.76) | <.001 |
| SOFA | 7.55 (4.03) | 6.90 (3.61) | 10.49 (4.49) | <.001 |
| APACHEII | 79.25 (28.73) | 74.86 (25.60) | 99.19 (33.38) | <.001 |
| ICU day, day | 5.58 (6.63) | 5.44 (6.47) | 6.24 (7.30) | .009 |
| Hospital dead, n (p%) |  |  |  | <.001 |
| No | 2,826 (74.1) | 2,819 (90.2) | 7 (1.0) |  |
| Yes | 989 (25.9) | 308 (9.8) | 681 (99.0) |  |

**Table S4.** Baseline characteristics of SIC patients in Tianjin Medical University General Hospital.

| **Variable** | **Overall** | **Survival** | **Death** | ***P* value** |
| --- | --- | --- | --- | --- |
|  | N = 235 | N = 190 | N = 45 |  |
| Age, year | 68.06 (15.63) | 66.86 (16.15) | 73.37 (11.91) | .004 |
| RDW, % | 17.90 (4.30) | 17.91 (4.37) | 17.89 (4.05) | .98 |
| Anion gap, mmol/l | 11.58 (4.32) | 11.40 (4.24) | 12.35 (4.65) | .24 |
| Lactate, mmol/L | 1.72 (1.12) | 1.65 (0.98) | 2.02 (1.57) | .16 |
| PTT, seconds | 35.21 (12.94) | 34.28 (12.46) | 39.29 (14.36) | .04 |
| BUN, mg/dL | 28.63 (19.59) | 27.83 (18.74) | 32.14 (22.91) | .27 |
| Total bilirubin, mg/dL | 16.03 (19.77) | 15.35 (17.86) | 19.03 (26.65) | .40 |
| Heart rate, times/minute | 92.07 (21.47) | 89.83 (21.16) | 101.93 (20.24) | .001 |
| SpO_2_, % | 92.19 (5.22) | 92.63 (4.69) | 90.24 (6.81) | .04 |
| Vasopressor, n (p%) |  |  |  | .06 |
| No | 192 (86.9) | 160 (88.9) | 32 (78.0) |  |
| Yes | 29 (13.1) | 20 (11.1) | 9 (22.0) |  |
| SIC_score, n (p%) |  |  |  | .002 |
| 4 | 57 (25.8) | 53 (29.5) | 4 (9.8) |  |
| 5 | 56 (25.3) | 49 (27.2) | 7 (17.1) |  |
| 6 | 108 (48.9) | 78 (43.3) | 30 (73.1) |  |
| SOFA | 4.86 (3.38) | 4.29 (2.79) | 7.32 (4.51) | <.001 |
| APACHEII | 14.74 (7.59) | 13.33 (6.41) | 20.93 (9.22) | <.001 |

**Table S5.** Results of the multivariable logistic regression model for feature selection.

| **Variable** | ***P* value** | **OR** | **Lower_OR** | **Upper_OR** |
| --- | --- | --- | --- | --- |
| **Age** | <.001 | 1.026 | 1.021 | 1.031 |
| PNA | .06 | 1.640 | 1.420 | 1.893 |
| CVA | .06 | 1.306 | 1.050 | 1.619 |
| MI | .12 | 1.345 | 1.055 | 1.708 |
| RDW | <.001 | 1.144 | 1.114 | 1.174 |
| Anion_gap | <.001 | 1.046 | 1.023 | 1.070 |
| Lactate | <.001 | 1.129 | 1.085 | 1.176 |
| PT | .24 | 1.004 | 0.997 | 1.010 |
| PTT | <.001 | 1.008 | 1.004 | 1.011 |
| Total_bilirubin | <.001 | 1.032 | 1.021 | 1.044 |
| Creatinine | .08 | 0.864 | 0.808 | 0.921 |
| BUN | <.001 | 1.011 | 1.007 | 1.014 |
| crrt | .05 | 2.082 | 1.673 | 2.594 |
| Heart_rate | <.001 | 1.011 | 1.007 | 1.016 |
| SpO_2_ | <.001 | 0.895 | 0.867 | 0.923 |
| Vasopressor | <.001 | 2.667 | 2.248 | 3.174 |

**Table S6.** VIF values for the final selected predictors.

| **Variable** | **VIF** |
| --- | --- |
| Age | 1.223 |
| RDW | 1.132 |
| Anion gap | 1.879 |
| Lactate | 1.571 |
| PTT | 1.029 |
| Total bilirubin | 1.264 |
| BUN | 1.404 |
| Heart rate | 1.108 |
| SpO_2_ | 1.034 |
| Vasopressor | 1.035 |

**Table S7.** Performance metrics of the XGBoost model in the two external validation sets.

|  | **Accuracy** | **Recall** | ***F*_1_-Score** | **AUROC** | **Specificity** | **FNR** | **FPR** |
| --- | --- | --- | --- | --- | --- | --- | --- |
| External validation set one | 0.805 | 0.797 | 0.803 | 0.882 | 0.813 | 0.203 | 0.187 |
| External validation set two | 0.815 | 0.811 | 0.811 | 0.904 | 0.818 | 0.189 | 0.182 |

**Table S8.** Evaluation metrics of machine learning models for in-hospital mortality prediction.

| **Model Name** | **Accuracy** | **Recall** | ***F*_1_-Score** | **AUROC** | **Specificity** | **FNR** | **FPR** |
| --- | --- | --- | --- | --- | --- | --- | --- |
| **XGBoost** | **0.906** | **0.900** | **0.907** | **0.963** | **0.912** | **0.100** | **0.088** |
| KNN | 0.813 | 0.984 | 0.843 | 0.926 | 0.634 | 0.016 | 0.366 |
| ANN | 0.852 | 0.934 | 0.866 | 0.916 | 0.767 | 0.066 | 0.233 |
| SVM | 0.628 | 0.707 | 0.659 | 0.665 | 0.547 | 0.293 | 0.453 |
| CatBoost | 0.833 | 0.856 | 0.839 | 0.906 | 0.809 | 0.144 | 0.191 |
| NB | 0.544 | 0.443 | 0.498 | 0.574 | 0.649 | 0.557 | 0.351 |
| RF | 0.637 | 0.495 | 0.581 | 0.730 | 0.784 | 0.505 | 0.216 |
| Logistic | 0.642 | 0.629 | 0.642 | 0.690 | 0.654 | 0.371 | 0.346 |
| LGBM | 0.876 | 0.880 | 0.879 | 0.947 | 0.873 | 0.120 | 0.127 |
| AdaBoost | 0.777 | 0.810 | 0.787 | 0.776 | 0.742 | 0.190 | 0.258 |
| GBDT | 0.778 | 0.817 | 0.789 | 0.817 | 0.738 | 0.183 | 0.262 |
| DecisionTree | 0.640 | 0.482 | 0.577 | 0.703 | 0.804 | 0.518 | 0.196 |

**Table S9.** Detailed performance metrics of the XGBoost model using 10-fold cross-validation.

| **Model Name** | **Accuracy** | **Recall** | ***F*_1_-Score** | **AUROC** | **Specificity** | **FNR** | **FPR** |
| --- | --- | --- | --- | --- | --- | --- | --- |
| XGBoost_1 | 0.829 | 0.836 | 0.833 | 0.903 | 0.822 | 0.164 | 0.178 |
| XGBoost_2 | 0.835 | 0.832 | 0.838 | 0.903 | 0.839 | 0.168 | 0.161 |
| XGBoost_3 | 0.826 | 0.83 | 0.825 | 0.91 | 0.823 | 0.17 | 0.177 |
| XGBoost_4 | 0.834 | 0.849 | 0.837 | 0.907 | 0.818 | 0.151 | 0.182 |
| XGBoost_5 | 0.832 | 0.842 | 0.828 | 0.902 | 0.823 | 0.158 | 0.177 |
| XGBoost_6 | 0.81 | 0.813 | 0.812 | 0.891 | 0.806 | 0.187 | 0.194 |
| XGBoost_7 | 0.829 | 0.824 | 0.829 | 0.892 | 0.834 | 0.176 | 0.166 |
| XGBoost_8 | 0.815 | 0.834 | 0.82 | 0.89 | 0.796 | 0.166 | 0.204 |
| XGBoost_9 | 0.836 | 0.845 | 0.833 | 0.904 | 0.828 | 0.155 | 0.172 |
| XGBoost_10 | 0.825 | 0.84 | 0.83 | 0.899 | 0.809 | 0.16 | 0.191 |

**Figure S1.** ROC curves of the XGBoost model using 10-fold cross-validation.

**Table S10.** Logistic regression analysis of predicted models for 28-day mortality in MIMIC-IV.

| **Model** | **OR (univariable)** | **OR (multivariable)** |
| --- | --- | --- |
| XGBoost (mean ± SD) | 47.96 (37.43-61.81,  P<.001) | 31.45 (24.05-41.35,  P<.001) |
| SOFA (mean ± SD) | 1.19 (1.17-1.21,  P<.001) | 0.99 (0.97-1.02,  *P*=.67) |
| APACHEII (mean ± SD) | 1.11 (1.10-1.12,  P<.001) | 1.00 (0.98-1.02,  P=.94) |
| SAPSII (mean ± SD) | 1.06 (1.05-1.07,  P<.001) | 1.02 (1.01-1.03,  P<.001) |
| OASIS (mean ± SD) | 1.08 (1.07-1.09,  P<.001) | 1.04 (1.02-1.05,  P<.001) |

**Table S11.** Logistic regression analysis of predicted models for 28-day mortality in eICU-CRD.

| **Model** | **OR (univariable)** | **OR (multivariable)** |
| --- | --- | --- |
| XGBoost (mean ± SD) | 77.47 (54.98-110.57,  P<.001) | 52.05 (36.23-75.7,  P<.001) |
| SOFA (mean ± SD) | 1.24 (1.21-1.28,  P<.001) | 1.01 (0.97-1.06,  *P*=.55) |
| APACHEII (mean ± SD) | 1.03 (1.02-1.04,  P<.001) | 1.01 (1.00-1.02,  P=.04) |
| SAPSII (mean ± SD) | 1.06 (1.05-1.07,  P<.001) | 1.02 (1.00-1.03,  P=.01) |
| OASIS (mean ± SD) | 1.08 (1.07-1.09,  P<.001) | 1.03 (1.01-1.05,  P<.001) |

**Table S12.** Logistic regression analysis of predicted models for 28-day mortality in Tianjin Medical University General Hospital.

| **Model** | **OR (univariable)** | **OR (multivariable)** |
| --- | --- | --- |
| XGBoost (mean ± SD) | 122.34 (30.21-649.17,  P<.001) | 113.88 (24.32-762.29,  P<.001) |
| SOFA (mean ± SD) | 1.28 (1.13-1.49,  P<.001) | 1.04 (0.83-1.36,  P<.001) |
| APACHEII (mean ± SD) | 1.14 (1.08-1.22,  P<.001) | 1.12 (1.00-1.26,  P<.001) |
